# Supplementary material for: Specific Determinants of the Transmembrane Region of the Andes Virus Gc Glycoprotein Drive the Transition from Membrane Hemifusion to Pore Formation
Source: Viruses. 2026 May 31;18(6):633. doi: 10.3390/v18060633 (PMC13307830; doi:10.3390/v18060633)
Supplement: Supplementary file 1 [file viruses-18-00633-s001.zip › viruses-4235836-supplementary.pdf]

A)

```
                         Gc-TMD
ANDV     GILNGNWIVVVVLVVILILSIIMFSVLCPRRGHKKTV---  1138
SNV      GILNGNWVVVAVLIVILILSILLFSFFCPVRSRKNKAN--  1140
PUUV     GVLNGNWMVVAVLVVLLILSILLFTLCCPRRPSYRKEHKP  1148
DOBRV    GIFNGNWIVIVVLVFFFILSLILLSLLCPIRKHKRS----  1135
SEOV     GIINGNWVVLIVLCVLLLFSLILLSILCPVRKHKKS----  1133
HANTV    GIFSGNWIVLIVLCVFLLFSLVLLSILCPVRKHKKS----  1135
         *::.***:*: ** .::::*:::::. ** *    ..
```

B)

```
ANDV     LGILNGNWIVVVVLVVILILSIIMFSVLCPRRGHKKTV-------------------1138
TPMV     MGILSGNWLVIVVLVVIMILSIMLLSFFCPSKKHQA---------------------1121
BRNV     KGLFSGNWLVLILMLVLLIGSLFLLILLCPVKKYRN---------------------1136
LAIV     GGLFRGNWWVLLVLVGITILSILLLSMLCPAHRK-----------------------1127
HOLGV    KGLFSGNFFLPLILIGIALFSIILLSFLLPARRRT----------------------1123
WEMBV    DKIRSQPWWVWFILAI-AILVVILGLIACSCIIRAKREYTLLKER------------1228
WEHV     DSAVNGGVIAAIVILVAAIISMIVIYVVCRCSKKKAREDDTMLR--KISKKINKSQ-1192
WEYGV    VGFFENGWWIFVLVAIAALIVLMMV-RRCMNGRKSVWEGMTPSERKEISKKLFEKMA1140
                    .::    :  :::
```

**Figure S1.** Multiple amino acid sequence alignment of the hantavirus glycoprotein precursor protein GPC from representative hantaviruses. Conserved residues are labeled below through point scheme; “:” indicates strongly conserved substitution while “.” indicates weakly conserved substitution. The predicted ANDV TMD sequence is underlined. Positively charged residues are indicated in blue, negatively charged residues in red. The conserved serine residues S1121 and S1126 (numeration from ANDV GPC) are indicated in bold letters. Numbers at the end of the alignment indicate the length of each respective GPC. **A)** Sequence alignment from GPC of representative pathogenic hantaviruses from the Orthohantavirus genus. ANDV (Andes virus, GenBank AAO86638.1), SNV (Sin Nombre virus, GenBank AAA75530), PUUV (Puumala virus, GenBank P21400, DOBRV (Dobrava virus NP_942554), SEOV (Seoul virus, GenBank NP_942557), HTNV (Hantaan virus, GenBank P08668. **B)** Sequence alignment from one representative protype of each of the eight genera of the Hantavirdae family. ANDV (Andes virus, Orthohantavirus genus (mammal hosts), GenBank: AAO86638.1), TPMV (Thottapalayam virus, Thottimvirus genus (mammal hosts), GenBank ABH09887.1), BRNV (Brno virus, Loanvirus genus (mammal hosts), GenBank APU53640.1), LAIV (Laibin virus, Mobatvirus genus (mammal hosts), Genbank AJZ68871.1), HOLGV (Hainan oriental leaf-toed gecko hantavirus, *Reptillovirus* genus (reptilian hosts), GenBank AVM87654.1), WEMBV (Wenling minipizza batfish hantavirus, *Actinovirus* genus (piscine hosts), GenBank: AVM87657.1), WEHV (Wenling hagfish virus, *Agnathovirus* genus, (piscine hosts), GenBank AVM87666.1), WEYGV (Wenling yellow goosefish hantavirus, *Perciloirus* genus (piscine hosts), GenBank AVM87660.1).

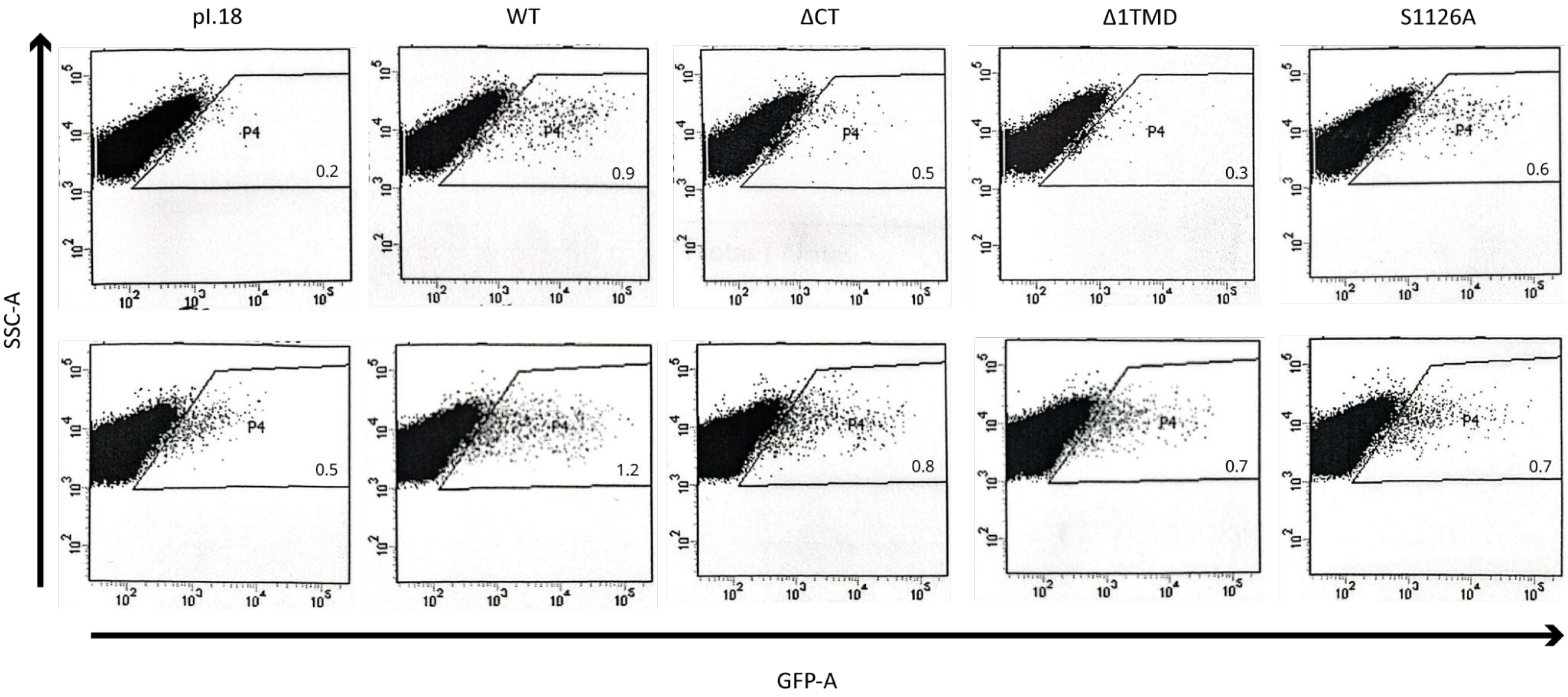


**Figure S2.** Raw data of cell cytometry dot blots of Vero E6 cells transduced with SIV vectors pseudotyped with ANDV WT or TMD mutants. SIV vectors pseudotyped with ANDV Gc WT or TMD were used to transduce Vero E6 and a GFP reporter gene expression measured 72 h post-transduction. Gates showing the percentage of cells expressing GFP were established using untransfected cells and cells transfected with the empty vector (pI.18) to rule out autofluorescence. Dot blots were acquired from N = 2 independent experiments. For the calculation of positive events, the negative control signal from pI.18 transfected cells was subtracted from the experimental samples to account for background fluorescence.
